# Synthesis of Pyrrolo[2,1-*a*]isoquinolines by Multicomponent 1,3-Dipolar Cycloaddition

**DOI:** 10.3390/molecules18032635

**Published:** 2013-02-27

**Authors:** Florea Dumitrascu, Emilian Georgescu, Florentina Georgescu, Marcel Mirel Popa, Denisa Dumitrescu

**Affiliations:** 1 Center of Organic Chemistry “C. D. Nenitzescu”, Romanian Academy, Spl. Independentei 202B, Bucharest 060023, Romania; E-Mail: fdumitra@yahoo.com (F.D.); 2 Research Center Oltchim, St. Uzinei 1, Ramnicu Vilcea 240050, Romania; E-Mail: emilian.georgescu@oltchim.com (E.G.); 3 Faculty of Pharmacy, “Ovidius” University, Aleea Universitatii nr.1, Campus Corp B, Constantza 900470, Romania

**Keywords:** pyrrolo[2,1-*a*]isoquinoline, one-pot three component, 1,3-dipolar cycloaddition

## Abstract

Pyrrolo[2,1-*a*]isoquinoline derivatives were synthesized by one-pot three-component reactions starting from isoquinoline, 2-bromoacetophenones and different non-symmetrical acetylenic dipolarophiles using 1,2-epoxypropane as solvent. The structure of the compounds was assigned by IR and NMR spectroscopy.

## 1. Introduction

**Figure 1:**
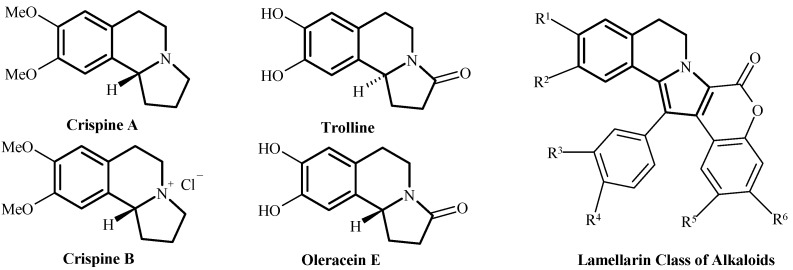
Natural alkaloids with pyrrolo[1,2-*a*]isoquinoline core.

Pyrrolo[2,1-*a*]isoquinolines are *N*-bridgehead heterocyclic compounds which are structural elements of natural products (Figure 1) of great significance for their biological activity, such as crispine A (Figure 1), with important anticancer activity [1,2,3,4,5]. Recently studied natural products with pyrrolo[2,1-*a*]isoquinoline cores are oleracein E [6,7] and trolline [8] (Figure 1), which were isolated from traditional Chinese medicinal plants. 

Maybe one of the most important classes of natural compounds are the lamellarins (Figure 1), which are known to posses an array of biological properties such as cell differentiation inhibition and cytotoxicity [9,10,11,12,13], this leading to numerous studies on lead compounds with analogous structures [14].

In this regard efforts were directed to synthesize aromatic or hydrogenated pyrrolo[2,1-*a*]-isoquinoline frameworks in the search for molecules relevant for medicinal purposes. The synthesis and properties of the pyrrolo[2,1-*a*]isoquinolines were reviewed in 1997 by Mikhailovskii and Shklyaev [15], but the synthesis and characterization of these compounds is still of current interest, the proof being the important number of very recently reported papers [16,17,18,19].

One of the important and current methods for the synthesis of pyrrolo[2,1-*a*]isoquinolines is the 1,3-dipolar cycloaddition reaction of isoquinolinium *N*-ylides with activated alkynes or olefins [20,21,22,23,24,25,26,27,28]. Our interest in studying convenient and simple methods for obtaining new pyrroloazine derivatives [29,30,31,32,33,34] led us to expand our studies to pyrrolo[2,1-*a*]isoquinolines [27]. The success in synthesis of such compounds by two methods involving two step procedures [27] led us to examine the one-pot three component procedure for the synthesis of pyrrolo[2,1-*a*]isoquinoline derivatives by 1,3-dipolar reactions discussed herein. The key components are isoquinoline, substituted bromoacetophenones and activated acetylenic dipolarophiles which react in 1,2-epoxypropane to yield the desired products with high efficiency.

## 2. Results and Discussion

**Scheme 1:**
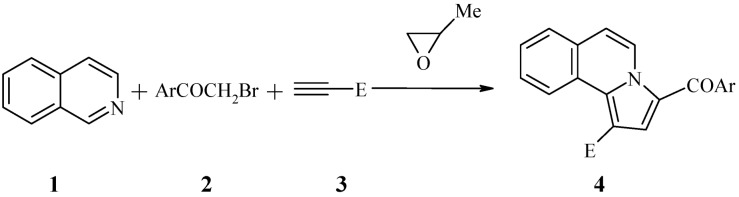
The one-pot three component synthesis of the new compounds.

Syntheses involving multicomponent one-pot reactions have provided useful synthetic tools in obtaining a wide variety of heterocyclic systems [35,36,37]. Thus a 1,3-dipolar cycloaddition targeting pyrrolo[2,1-*a*]isoquinoline derivatives, conducted as a one-pot three component process, seemed to be a very promising route. The key components of the one-pot three component reaction for the synthesis of pyrrolo[2,1-*a*]isoquinolines **4** (Table 1) are isoquinoline (**1**), the substituted bromoacetophenones **2**, the non-symmetrical electron deficient alkynes **3** and 1,2-epoxypropane which acts both as solvent and proton scavenger (Scheme 1). Using this methodology the series of compounds listed in Table 1 was prepared in fair to good yields.

**Table 1:** New pyrrolo[2,1-*a*]isoquinolines **4**.

No.	R	E	Ar	M.p. (°C)	Yield (%)
**4a**	H	COMe	4-MeOC_6_H_4_	171–173	71
**4b**	H	COMe	3-NO_2_C_6_H_4_	218–220	70
**4c**	H	COMe	3,4-(MeO)_2_C_6_H_3_	198–200	65
**4d**	H	CO_2_Me	2-ClC_6_H_4_	222–225	60
**4e**	H	CO_2_Me	2,4-Cl_2_C_6_H_3_	205–208	69
**4f**	H	CO_2_Me	3-NO_2_C_6_H_4_	209–212	70
**4g**	H	CO_2_Me	4-NO_2_C_6_H_4_	208–211	64
**4h**	H	CO2Et	1-naphthyl	162–164	72
**4i**	H	CO_2_Et	2-napthyl	150–152	67
**4j**	H	CO2Et	2-NO_2_C_6_H_4_	186–187	69
**4k**	H	CO_2_Et	3-NO_2_C_6_H_4_	201–203	78
**4l**	H	CO_2_Et	4-NO_2_C_6_H_4_	209–211	63
**4m**	H	CO_2_Et	4-FC_6_H_4_	140–142	65
**4n**	H	CO_2_Et	2,4-Cl_2_C_6_H_3_	180–186	52
**4o**	H	CO_2_Et	4-BrC_6_H_4_	190–192	66
**4p**	H	CO_2_Et	2-HOC_6_H_4_	152–154	64
**4q**	H	CO_2_Et	4-MeOC_6_H_4_	151–153	61
**4r**	H	CO_2_Et	3,4-(MeO)_2_C_6_H_3_	173–176	69

**Scheme 2:**
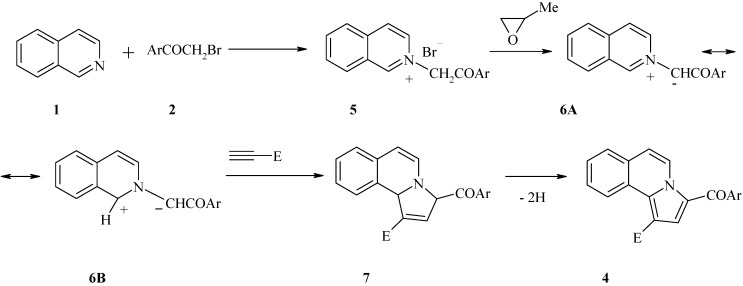
Reaction mechanism.

The reaction mechanism (Scheme 2) for formation of the pyrroloisoquinolines **4** involves in the first step the generation of isoquinolinium *N*-ylides **6A** by the action the isoquinolinium bromides **5** on the epoxide which, on nucleophilic ring opening by bromide anion, generates an alkoxide for deprotonation of the salt to form **6A**. Subsequently, the 1,3-dipolar cycloaddition between the 1,3-dipole **6B** and the unsymmetrical acetylenic dipolarophiles afford the corresponding primary cycloadducts **7** which undergoes a spontaneous *in situ* rearrangement and dehydrogenation leading to the fully aromatic compounds **4**.

It is important to mention that no hydrogenated intermediates were isolated as for the previously reported two step procedure [27]. By comparison with the two step procedure the yields are appreciably lower but this minor inconvenience is significantly overcome by the more simple procedure and economy of both time and materials.

The structures of the new pyrroloisoquinolines were assigned by IR and NMR spectroscopy. The FT-IR spectra of the compounds present the characteristic bands for carbonyl groups that appear in the expected ranges, and the characteristic bands for the particular functional groups present in each example are also observed. On the basis of NMR data it was found that the cycloaddition reaction between isoquinolinium *N*-ylides and unsymmetrical dipolarophile is completely regioselective, as only one regioisomer was obtained. This is proven by the signal of the H-2 hydrogen which appears as a sharp singlet.

In the ^1^H-NMR spectra of compounds **4** the general characteristic features are the chemical shifts of atoms H-5, H-6 and H-10. The two protons in the pyridine moiety, namely H-5 and H-6, appear as two doublets with a coupling constant of 7.4 Hz. The H-10 hydrogen appears as a deshielded multiplet due to the spatial vicinity with the carbonyl group in the acetyl or ester groups. The ^13^C-NMR spectra show all the expected signals. The most characteristic feature is the strong shielding observed for C-1 which appears at around 110 ppm as a consequence of its relative β position with respect to the pyrrole nitrogen. For the compounds **4a**–**c** the carbon C-1 appears slightly deshielded to 118 ppm due to the influence of an acetyl group instead of an ester group. The carbon atoms in the carbonyl groups were observed in the expected ranges.

## 3. Experimental

### 3.1. General

Melting points were determined on a Boëtius hot plate microscope and are uncorrected. The elemental analysis was carried out on a COSTECH Instruments EAS32 apparatus. The IR spectra were recorded on a FT-IR Bruker Vertex 70. The NMR spectra were recorded on a Varian Gemini 300 BB instrument, operating at 300 MHz for ^1^H-NMR and 75 MHz for ^13^C-NMR. Supplementary evidence was given by HETCOR and COSY experiments. 

### 3.2. General Procedure for the Synthesis of pyrrolo[2,1-a]isoquinolines **4**

Isoquinoline (**1**, 3 mmol), phenacyl bromide **2** (3 mmol) and the corresponding acetylenic dipolarophile (2-butyn-3-one, methyl propiolate, ethyl propiolate) **3** (5 mmol) in 1,2-epoxypropane (15 mL) were stirred at reflux for 20 h. The solvent was partly removed by evaporation, methanol or ethanol (10 mL) was added and the mixture was left overnight in the refrigerator. The solid formed was filtered, washed with ethanol and crystallized from CHCl_3_/MeOH.

*1-Acetyl-3-(4-methylbenzoyl)-pyrrolo[2,1-a]isoquinoline* (**4a**). Light yellow crystals, m.p. 171–173 °C; Yield 71%. Anal. Calcd. C_22_H_17_NO_2_: C 80.71, H 5.23, N 4.28; Found: C 80.51, H 5.02, N 4.57. FT-IR (cm^−1^): 1172, 1332, 1359, 1444, 1517, 1619, 1655, 2920. ^1^H-NMR (CDCl_3_) δ: 2.47, 2.64 (2s, 6H, 2Me); 7.29 (d, 1H, *J* = 7.4 Hz, H-6); 7.35 (d, 2H, *J* = 8.0 Hz, H-3', H-5'); 7.64–7.68 (m, 2H, H-7, H-8); 7.71 (s, 1H, H-2); 7.73–7.75 (m, 1H, H-9); 7.78 (d, 2H, *J* = 8.0 Hz, H-2', H-6'); 9.60 (d, 1H, *J* = 7.4 Hz, H-5); 9.80–9.82 (m, 1H, H-10). ^13^C-NMR (CDCl_3_) δ: 21.7, 30.1 (2Me); 116.2 (C-6); 119.2 (C-1); 123.7, 124.9, 130.9, 137.2 (C-3, C-6a, C-10a, C-10b); 125.0 (C-5); 126.7, 127.8, 129.7, 129.9 (C-2, C-7, C-8, C-9); 128.4 (C-10); 129.3, 129.5 (C-2', C-3', C-5', C-6'); 136.5 (C-1'); 142.7 (C-4'); 185.9 (COAr); 193.7 (COO).

*1-Acetyl-3-(3-nitrobenzoyl)-pyrrolo[2,1-a]isoquinoline* (**4b**). Light yellow crystals, m.p. 218–220 °C; Yield 70%. Anal. Calcd. C_21_H_14_N_2_O_4_: C 70.39, H 3.94, N 7.82; Found: C 70.65, H 3.72, N 8.08. FT-IR (cm^−1^): 1180, 1339, 1446, 1514, 1615, 1666, 3004. ^1^H-NMR (CDCl_3_) δ: 2.64 (s, 3H, Me); 7.33 (d, 1H, *J* = 7.4 Hz, H-6); 7.66–7.77 (m, 4H, H-7, H-8, H-9, H-5'); 7.65 (s, 1H, H-2); 8.15–8.19 (m, 1H, H-6'); 8.43–8.47 (m, 1H, H-4'); 8.68 (t, 1H, *J* = 1.9 Hz, H-2'); 9.60 (d, 1H, *J* = 7.4 Hz, H-5); 9.73–9.76 (m, H, H-10). ^13^C-NMR (CDCl_3_) δ: 30.0 (Me); 116.8 (C-6); 119.9 (C-1); 123.9 (C-2'); 122.5, 124.6, 130.9, 137.1 (C-3, C-6a, C-10a, C-10b); 124.6 (C-5); 126.7, 128.0, 129.7, 130.1 (C-2, C-7, C-8, C-9); 128.4 (C-10); 129.7, 130.2, 134.6 (C-4', C-5', C-6'); 141.3 (C-1'); 148.2 (C-3'); 182.7 (COAr); 193.4 (COO).

*1-Acetyl-3-(3,4-dimethoxylbenzoyl)-pyrrolo[2,1-a]isoquinoline* (**4c**). Light yellow crystals, m.p. 198–200 °C; Yield 65%. Anal. Calcd. C_23_H_19_NO_4_: C 73.98, H 5.13, N 3.75; Found: C 74.31, H 5.37, N 4.06. FT-IR (cm^−1^): 1175, 1269, 1360, 1451, 1512, 1623, 1665, 2966. ^1^H-NMR (CDCl_3_) δ: 2.65 (s, 3H, Me); 3.97, 3.98 (2s, 6H, 2MeO); 6.97 (d, 1H, *J* = 8.2 Hz, H-5'); 7.22 (d, 1H, *J* = 7.4 Hz, H-6); 7.48–7.51 (m, 2H, H-2', H-6'); 7.60–7.71 (m, 3H, H-7, H-8, H-9); 7.72 (s, 1H, H-2); 9.46 (d, 1H, *J* = 7.4 Hz, H-5); 9.77–9.80 (m, 1H, H-10). ^13^C-NMR (CDCl_3_) δ: 30.0 (2Me); 29.9 (Me); 56.1 (2MeO); 110.1, 111.9 (C-2', C-5'); 115.9 (C-6); 118.9 (C-1); 123.5, 124.9, 130.5, 136.1 (C-3, C-6a, C-10a, C-10b); 123.7 (C-6'); 124.6 (C-5); 126.6, 127.6, 129.4, 129.5 (C-2, C-7, C-8, C-9); 128.2 (C-10); 132.3 (C-1'); 149.1, 152.6 (C-3', C-4'); 184.5 (COAr); 193.4 (COO).

*Methyl 3-(2-chlorobenzoyl)-pyrrolo[2,1-a]isoquinoline-1-carboxylate* (**4d**). Light yellow crystals, m.p. 225–258 °C; Yield 60%. Anal. Calcd. C_21_H_14_ClNO_3_: C 69.33, H 3.88, Cl 9.75, N 3.85; Found: C 69.59, H 3.61, Cl 9.47, N 4.14. FT-IR (cm^−1^): 1181, 1339, 1453, 1524, 1629, 1704, 2951. ^1^H-NMR (CDCl_3_) δ: 3.87 (s, 3H, Me); 7.30 (d, 1H, *J* = 7.4 Hz, H-6); 7.39–7.51 (m, 4H, H-7, H-8, H-3', H-5'); 7.54 (s, 1H, H-2); 7.64–7.67 (m, 2H, H-9, H-4'); 7.74–7.77 (m, 1H, H-6'); 9.77–9.81 (m, 2H, H-5, H-10). ^13^C-NMR (CDCl_3_) δ: 51.8 (Me); 110.6 (C-1); 116.1 (C-6); 123.4, 124.5, 130.8, 137.5 (C-3, C-6a, C-10a, C-10b); 125.2 (C-5); 126.6, 129.2, 130.3, 131.1 (C-3', C-4', C-5', C-6'); 126.8, 127.9, 129.6, 130.9 (C-2, C-7, C-8, C-9); 128.3 (C-10); 131.5, 139.6 (C-1', C-2'); 164.8 (COO); 184.1 (COAr).

*Methyl 3-(3-nitrobenzoyl)-pyrrolo[2,1-a]isoquinoline-1-carboxylate* (**4e**). Light yellow crystals, m.p. 190–192 °C; Yield 69%. Anal. Calcd. C_21_H_14_N_2_O_5_: C 67.38, H 3.77, N 7.48. Found: C 67.57, H 3.51, N 7.69. FT-IR (cm^−1^): 1183, 1339, 1459, 1527, 1631, 1712, 2956. ^1^H-NMR (CDCl_3_) δ: 3.90 (s, 3H, Me); 7.30 (d, 1H, *J* = 7.4 Hz, H-6); 7.66–7.78 (m, 4H, H-7, H-8, H-9, H-5'); 7.74 (s, 1H, H-2); 8.13–8.16 (m, 1H, H-6'); 8.41–8.45 (m, 1H, H-4'); 8.66 (t, 1H, *J* = 1.9 Hz, H-2'); 9.61 (d, 1H, *J* = 7.4 Hz, H-5); 9.81–9.84 (m, H, H-10). ^13^C-NMR (CDCl_3_) δ: 52.0 (Me); 110.7 (C-1); 116.3 (C-6); 122.6, 124.6, 130.8, 137.8 (C-3, C-6a, C-10a, C-10b); 124.1 (C-2'); 125.0 (C-5); 126.9, 129.8, 134.8 (C-4', C-5', C-6'); 126.1, 128.3, 129.9, 130.5 (C-2, C-7, C-8, C-9); 128.4 (C-10); 141.5 (C-1'); 148.3 (C-3'); 164.7 (COO); 183.0 (COAr). 

*Methyl 3-(4-nitrobenzoyl)-pyrrolo[2,1-a]isoquinoline-1-carboxylate* (**4f**). Light yellow crystals, m.p. 209–212 °C; Yield 70%. Anal. Calcd. C_21_H_14_N_2_O_5_: C 67.38, H 3.77, N 7.48. Found: C 67.21, H 4.02, N 7.71. FT-IR (cm^−1^): 1183, 1339, 1453, 1521, 1617, 1711, 2957. ^1^H-NMR (CDCl_3_) δ: 3.92 (s, 3H, Me); 7.33 (d, 1H, *J* = 7.4 Hz, H-6); 7.67–7.80 (m, 3H, H-7, H-8, H-9); 7.74 (s, 1H, H-2); 7.95 (d, 2H, *J* = 8.8 Hz, H-2', H-6'); 7.95 (d, 2H, *J* = 8.8 Hz, H-3', H-5'); 9.66 (d, 1H, *J* = 7.4 Hz, H-5); 9.83–9.85 (m, 1H, H-10). ^13^C-NMR (CDCl_3_) δ: 52.0 (Me); 110.7 (C-1); 116.4 (C-6); 122.7, 124.5, 130.9, 137.8 (C-3, C-6a, C-10a, C-10b); 123.7 (C-2', C-6'); 125.0 (C-5); 126.9, 128.3, 129.9, 130.7 (C-2, C-7, C-8, C-9); 128.4 (C-10); 130.0 (C-3', C-5'); 145.4 (C-1'); 149.6 (C-3'); 164.7 (COO); 183.5 (COAr).

*Methyl 3-(2,4-dichlorobenzoyl)-pyrrolo[2,1-a]isoquinoline-1-carboxylate* (**4g**). Light yellow crystals, m.p. 205–208 °C; Yield 64%. Anal. Calcd. C_21_H_13_Cl_2_NO_3_: C 63.34, H 3.29, Cl 17.80, N 3.52. Found: C 63.59, H 3.51, Cl 18.07, N 3.81. FT-IR (cm^−1^): 1181, 1366, 1454, 1526, 1626, 1708, 2952. ^1^H-NMR (300 MHz, CDCl_3_) δ: 3.90 (s, 3H, Me); 7.34 (d, 1H, *J* = 7.4 Hz, H-6); 7.37–7.45 (m, 2H, H-5', H-6'); 7.53 (s, 1H, H-2); 7.54 (d, 1H, *J* = 1.7 Hz, H-3'); 7.66–7.71 (m, 2H, H-8, H-9); 7.76–7.80 (m, 1H, H-7); 9.75 (d, 1H, *J* = 7.4 Hz, H-5); 9.81–9.84 (m, 1H, H-10). ^13^C-NMR (75 MHz, CDCl_3_) δ: 52.0 (Me); 110.8 (C-1); 116.4 (C-6); 123.2, 124.6, 130.9, 132.7, 136.5, 137.8 (C-3, C-6a, C-10a, C-10b, C-1', C-2', C-4'); 125.2 (C-5); 126.9, 127.1, 128.2, 129.9, 130.2, 130.9, 131.0 (C-2, C-7, C-8, C-9, C-3', C-5', C-6'); 128.4 (C-10); 164.7 (COO); 182.9 (COAr).

*Ethyl 3-(1-Naphthoyl)-pyrrolo[2,1-a]isoquinoline-1-carboxylate* (**4h**). Light yellow crystals, m.p. 162–164 °C; Yield 72%. Anal. Calcd. C_26_H_19_NO_3_: C 79.37, H 4.87, N 3.56. Found: C 79.62, H 4.61, N 3.78. FT-IR (cm^−1^): 1186, 1357, 1460, 1527, 1615, 1714, 3039. ^1^H-NMR (CDCl_3_) δ: 1.31 (t, 3H, *J* = 7.1 Hz, Me); 4.32 (q, 2H, *J* = 7.1 Hz, CH_2_); 7.35 (d, 1H, *J* = 7.4 Hz, H-6); 7.49–7.59, 7.91–7.96, 8.14–8.20 (3m, 5H, H-3', H-5', H-6', H-7', H-8'); 7.62 (s, 1H, H-2); 7.66–7.69 (m, 2H, H-8, H-9); 7.72 (dd, 1H, *J* = 7.1, 1.3 Hz, H-4'); 7.77–7.82 (m, 1H, H-7); 8.02 (bd, 1H, H-2'); 9.79–9.84 (m, 1H, H-10); 9.92 (d, 1H, *J* = 7.4 Hz, H-5). ^13^C-NMR (CDCl_3_) δ: 14.3 (Me); 60.6 (CH_2_); 110.5 (C-1); 115.8 (C-6); 124.5, 124.6, 130.6, 130.9, 133.7, 137.2, 137.5 (C-3, C-6a, C-10a, C-10b, C-1', C-4a, C-8a); 125.3 (C-5); 124.4, 125.4, 126.4, 126.7, 126.9, 127.1, 127.8, 128.2, 130.7, 130.8 (C-7, C-8, C-9, C-2', C-3', C-4', C-5', C-6', C-7', C-8'); 128.3 (C-10); 129.4 (C-2); 164.5 (COO); 187.1 (COAr). 

*Ethyl 3-(2-Naphthoyl)-pyrrolo[2,1-a]isoquinoline-1-carboxylate* (**4i**). Beige crystals, m.p. 150–152 °C; Yield 67%. Anal. Calcd. C_26_H_19_NO_3_: C 79.37, H 4.87, N 3.56. Found: C 79.71, H 5.11, N 3.89. FT-IR (cm^−1^): 1184, 1361, 1452, 1524, 1611, 1704, 3057. ^1^H-NMR (CDCl_3_) δ: 1.37 (t, 3H, *J* = 7.1 Hz, Me); 4.38 (q, 2H, *J* = 7.1 Hz, CH_2_); 7.29 (d, 1H, *J* = 7.4 Hz, H-6); 7.58–7.70, 7.93–8.02 (2m, 8H, H-8, H-9, H-3', H-4', H-5', H-6', H-7', H-8'); 7.74–780 (m, 1H, H-7); 7.87 (s, 1H, H-2); 8.37 (bs, 1H, H-1'); 9.67 (d, 1H, *J* = 7.4 Hz, H-5); 9.82–9.87 (m, 1H, H-10). ^13^C-NMR (CDCl_3_) δ: 14.3 (Me); 60.5 (CH_2_); 110.2 (C-1); 115.4 (C-6); 123.4, 124.5, 130.4, 132.3, 134.8, 136.8, 137.0 (C-3a, C-6a, C-10a, C-10b, C-2', C-4a', C-8a'); 125.0 (C-5); 125.5, 126.6, 126.7, 127.6, 127.7, 127.8, 128.0, 129.1, 129.2, 129.9, 130.1 (C-2, C-7, C-8, C-9, C-1', C-3', C-4', C-5', C-6', C-7', C-8'); 128.2 (C-10); 164.5 (COO); 185.7 (COAr).

*Ethyl 3-(2-nitrobenzoyl)-pyrrolo[2,1-a]isoquinoline-1-carboxylate* (**4j**). Yellow crystals, m.p. 186–187 °C; Yield 69%. Anal. Calcd. C_22_H_16_N_2_O_5_: C 68.04, H 4.15, N 7.21. Found: C 68.41, H 4.47, N 7.47. FT-IR (cm^−1^): 1184, 1349, 1454, 1524, 1621, 1712, 2984. ^1^H-NMR (CDCl_3_) 1.35 (t, 3H, *J* = 7.1 Hz, Me); 4.35 (q, 2H, *J* = 7.1 Hz, CH_2_); 7.34 (d, 1H, *J* = 7.4 Hz, H-6); 7.41 (s, 1H, H-2); 7.62–7.73, 7.77–7.83 (2m, 6H, H-7, H-8, H-9, H-3', H-4', H-5'); 8.22–8.26 (m, 1H, H-6'); 9.74 (d, 1H, *J* = 7.4 Hz, H-5); 9.77–9.82 (m, 1H, H-10). ^13^C-NMR (CDCl_3_) δ: 14.5 (Me); 60.7 (CH_2_); 111.1 (C-1); 116.2 (C-6); 123.0, 124.7, 130.9, 136.2, 137.5 (C-3, C-6a, C-10a, C-10b, C-1'); 124.8 (C-5); 125.1 (C-3'); 126.9, 127.9, 129.0, 129.4, 129.6, 130.6, 133.2 (C-2, C-7, C-8, C-9, C-4', C-5', C-6'); 128.3 (C-10); 147.1 (C-2'); 164.5 (COO); 188.0 (COAr).

*Ethyl 3-(3-Nitrobenzoyl)-pyrrolo[2,1-a]isoquinoline-1-carboxylate* (**4k**). Yellow crystals, m.p. 203–205 °C; Yield 78%. Anal. Calcd. C_22_H_16_N_2_O_5_: C 68.04, H 4.15, N 7.21. Found: C 68.31, H 3.98, N 7.11. FT-IR (cm^−1^): 1189, 1349, 1455, 1531, 1625, 1720. ^1^H-NMR (CDCl_3_) δ: 1.40 (t, 3H, *J* = 7.1 Hz, Me); 4.41 (q, 2H, *J* = 7.1 Hz, CH_2_); 7.34 (d, 1H, *J* = 7.4 Hz, H-6); 7.67–7.83 (m, 4H, H-7, H-8, H-9, H-5'); 7.77 (s, 1H, H-2); 8.16–8.20, 8.44–8.48 (2m, 2H, H-4', H-6'); 8.70 (t, 1H, *J* = 1.8 Hz, H-2'); 9.66 (d, 1H, *J* = 7.4 Hz, H-5); 9.80–9.86 (m, 1H, H-10). ^13^C-NMR (CDCl_3_) δ: 14.6 (Me); 61.0 (CH_2_); 111.2 (C-1); 116.3 (C-6); 122.6, 124.6, 130.9, 137.8 (C-3a, C-6a, C-10a, C-10b); 124.1 (C-4'); 125.1 (C-5);127.0, 128.4, 129.9 (C-7, C-8, C-9); 126.2, 130.3 (C-2', C-5'); 128.2 (C-10); 129.8 (C-2); 134.8 (C-6') 141.5 (C-1'); 148.3 (C-3'); 164.4 (COO); 183.3 (COAr).

*Ethyl 3-(4-nitrobenzoyl)-pyrrolo[2,1-a]isoquinoline-1-carboxylate* (**4l**). Yellow crystals, m.p. 209–211 °C; Yield 63%. Anal. Calcd. C_22_H_16_N_2_O_5_: C 68.04; H 4.15; N 7.21. Found: C 68.37; H 4.51; N 7.61. FT-IR (cm^−1^): 1185, 1348, 1452, 1529, 1624, 1718. ^1^H-NMR (CDCl_3_) δ: 1.41 (t, 3H, *J* = 7.1 Hz, Me); 4.41 (q, 2H, *J* = 7.1 Hz, CH_2_); 7.34 (d, 1H, *J* = 7.4 Hz, H-6); 7.68–7.72 (m, 2H, H-8, H-9); 7.74 (s, 1H, H-2); 7.76–7.82 (m, 1H, H-7); 7.99 (d, 2H, *J* = 8.8 Hz, H-2', H-6'); 8.40 (d, 2H, *J* = 8.8 Hz, H-3', H-5'); 9.67 (d, 1H, *J* = 7.4 Hz, H-5); 9.80–9.86 (m, 1H, H-10). ^13^C-NMR (CDCl_3_) δ: 14.5 (Me); 61.0 (CH_2_); 111.0 (C-1); 116.3 (C-6); 123.8 (C-3', C-5'); 123.7, 124.5, 130.7, 137.6 (C-3a, C-6a, C-10a, C-10b); 125.1 (C-5); 126.9, 128.9, 129.8 (C-7, C-8, C-9); 128.4 (C-10); 129.7 (C-2); 130.1 (C-2', C-6'); 145.4 (C-1'); 149.6 (C-4'); 164.4 (COO); 183.5 (COAr).

*Ethyl 3-(4-fluorobenzoyl)-pyrrolo[2,1-a]isoquinoline-1-carboxylate* (**4m**). Yellow crystals, m.p. 141–143 °C; Yield 65%. Anal. Calcd. C_22_H_16_FNO_3_: N 3.88. Found: N 4.11. FT-IR (cm^−1^): 1182, 1360, 1457, 1619, 1709. ^1^H-NMR (CDCl_3_) 1.41 (t, 3H, *J* = 7.1 Hz, Me); 4.41 (q, 2H, *J* = 7.1 Hz, CH_2_); 7.18 (t, 2H, *J* = 8.8 Hz, H-2', H-6'); 7.21 (d, 1H, *J* = 7.4 Hz, H-6); 7.64–7.71 (m, 2H, H-8, H-9); 7.74 (s, 1H, H-2); 7.75–7.80 (m, 1H, H-7); 7.91 (dd, 2H, *J* = 8.8, 5.4 Hz, H-3', H-5'); 9.60 (d, 1H, *J* = 7.4 Hz, H-5); 9.80–9.85 (m, 1H, H-10). ^13^C-NMR (CDCl_3_) δ: 14.6 (Me); 60.8 (CH_2_); 110.5 (C-1); 115.7 (C-6); 115.8 (d, *J* = 21.8 Hz, C-3', C-5'); 123.3, 124.7, 130.7, 137.1 (C-3, C-6a, C-10a, C-10b); 125.1 (C-5); 126.8, 127.9, 129.8 (C-7, C-8, C-9); 128.3 (C-10); 129.5 (C-2); 131.8 (d, *J* = 9.2 Hz, C-2', C-6'); 136.2 (d, *J* = 2.9 Hz, C-1'); 164.7 (COO); 165.1 (d, *J* = 252.2 Hz, C-4'); 184.5 (COAr). 

*Ethyl 3-(2,4-dichlorobenzoyl)-pyrrolo[2,1-a]isoquinoline-1-carboxylate* (**4n**). Cream crystals, m.p. 184–186 °C; Yield 52%. Anal. Calcd. C_22_H_15_Cl_2_NO_3_: C 64.09; H 3.67; Cl 17.20, N 3.40. Found: C 64.44; H 4.02; Cl 17.51, N 3.21. FT-IR (cm^−1^): 1184, 1366. 1453, 1525, 1626, 1703, 2974. ^1^H-NMR (CDCl_3_) 1.39 (t, 3H, *J* = 7.1 Hz, Me); 4.39 (q, 2H, *J* = 7.1 Hz, CH_2_); 7.33 (d, 1H, *J* = 7.4 Hz, H-6); 7.39 (dd, 1H, *J* = 8.3, 1.8 Hz, H-5'); 7.44 (d, 1H, *J* = 8.3 Hz, H-6'); 7.52 (s, 1H, H-2); 7.54 (d, 1H, *J* = 1.8 Hz, H-3'); 7.65–7.70 (m, 2H, H-8, H-9); 7.75–7.80 (m, 1H, H-7); 9.77 (d, 1H, *J* = 7.4 Hz, H-5); 9.78–9.83 (m, 1H, H-10). ^13^C-NMR (CDCl_3_) δ: 14.5 (Me); 60.6 (CH_2_); 111.2 (C-1); 116.2 (C-6); 123.0, 124.5, 130.8, 132.6, 136.3, 137.6, 137.7 (C-3, C-6a, C-10a, C-10b, C-1', C-2', C-4'); 125.2 (C-5); 126.8, 127.0, 128.0, 130.1, 130.2, 130.7 (C-7, C-8, C-9, C-3', C-5', C-6'); 128.3 (C-10); 129.7 (C-2); 164.3 (COO); 184.9 (COAr).

*Ethyl 3-(4-bromobenzoyl)-pyrrolo[2,1-a]isoquinoline-1-carboxylate* (**4o**). Beige crystals, m.p. 190–192 °C; Yield 66%. Anal. Calcd. C_22_H_16_BrNO_3_: C 62.58; H 3.82; Br 18.92, N 3.32. Found: C 62.87; H 3.59; Br 19.31, N 3.63. FT-IR (cm^−1^): 1183, 1359, 1453, 1525, 1627, 1705, 2983. ^1^H-NMR (CDCl_3_) δ: 1.43 (t, 3H, *J* = 7.1 Hz, Me); 4.34 (q, 2H, *J* = 7.1 Hz, CH_2_); 7.23 (d, 1H, *J* = 7.4 Hz, H-6); 7.61–7.76 (m, 8H, H-2, H-7, H-8, H-9, H-2', H-3', H-5', H-6'); 9.55 (d, 1H, *J* = 7.4 Hz, H-5); 9.79–9.85 (m, 1H, H-10). ^13^C-NMR (CDCl_3_) δ: 14.6 (Me); 60.8 (CH_2_); 110.5 (C-1); 115.7 (C-6); 123.0, 124.5, 130.6, 137.1 (C-3, C-6a, C-10a, C-10b); 125.0 (C-5); 126.6 (C-4'); 126.8, 127.8, 129.7, 129.9 (C-2, C-7, C-8, C-9); 128.4 (C-10); 130.8, 131.7 (C-2', C-3', C-5', C-6'); 138.6 (C-4'); 164.4 (COO); 184.5 (COAr).

*Ethyl 3-(2-hydroxybenzoyl)-pyrrolo[2,1-a]isoquinoline-1-carboxylate* (**4p**). Yellow crystals, m.p. 152–154 °C; Yield 64%. Anal. Calcd. C_22_H_17_NO_4_: C 73.53; H 4.77; N 3.90. Found: C 73.88; H 4.46; N 4.21. FT-IR (cm^−1^): 1188, 1339, 1451, 1584, 1618, 1708, 2984. ^1^H-NMR (CDCl_3_) 1.42 (t, 3H, *J* = 7.1 Hz, Me); 4.43 (q, 2H, *J* = 7.1 Hz, CH_2_); 6.98–7.04 (m, 1H, H-5'); 7.07 (dd, 1H, *J* = 8.4, 1.1 Hz, H-3'); 7.26 (d, 1H, *J* = 7.4 Hz, H-6); 7.49–7.54 (m, 1H, H-4'); 7.61–7.67 (m, 2H, H-8, H-9); 7.71–7.77 (m, 1H, H-7); 7.87 (s, 1H, H-2); 7.90 (dd, 1H, *J* = 7.9, 1.6 Hz, H-6'); 9.21 (d, 1H, *J* = 7.4 Hz, H-5); 9.78–9.83 (m, 1H, H-10); 11.4 (s, 1H, OH). ^13^C-NMR (CDCl_3_) δ: 14.5 (Me); 60.6 (CH_2_); 110.7 (C-1); 115.4 (C-6); 118.3, 118.9 (C-2', C-5'); 120.9, 122.8 124.7, 130.5, 137.2 (C-3, C-6a, C-10a, C-10b, C-1'); 124.8 (C-5); 126.7, 127.9, 129.4, 129.6, 132.0, 135.2 (C-2, C-6, C-7, C-8, C-9, C-4', C-6'); 128.2 (C-10); 162.3 (C-2'); 164.5 (COO); 188.0 (COAr).

*Ethyl 3-(4-methoxybenzoyl)-pyrrolo[2,1-a]isoquinoline-1-carboxylate* (**4q**). Beige crystals, m.p. 147–149 °C; Yield 61%. Anal. Calcd. C_23_H_19_NO_4_: C 73.98; H 5.13; N 3.75. Found: C 74.29; H 4.89; N 4.11. FT-IR (cm^−1^): 1192, 1262, 1369, 1456, 1530, 1624, 1713, 2975. ^1^H-NMR (CDCl_3_) δ: 1.41 (t, 3H, *J* = 7.1 Hz, Me); 3.92 (s, 3H, MeO); 4.41 (q, 2H, *J* = 7.1 Hz, CH_2_); 7.04 (d, 2H, *J* = 8.8 Hz, H-3', H-5'); 7.35 (d, 1H, *J* = 7.4 Hz, H-6); 7.62–7.68 (m, 2H, H-8, H-9); 7.73–7.78 (m, 1H, H-7); 7.81 (s, 1H, H-2); 7.90 (d, 2H, *J* = 8.8 Hz, H-2', H-6'); 9.55 (d, 1H, *J* = 7.4 Hz, H-5); 9.79–9.85 (m, 1H, H-10). ^13^C-NMR (CDCl_3_) δ: 14.5 (Me); 55.6 (MeO); 60.6 (CH_2_); 111.0 (C-1); 113.7 (C-3', C-5'); 115.3 (C-6); 123.7, 124.5, 130.5, 136.7 (C-3, C-6a, C-10a, C-10b); 125.1 (C-5); 126.8, 127.8, 129.1 (C-7, C-8, C-9); 128.1 (C-10); 129.2 (C-2); 131.6 (C-2', C-6'); 132.4 (C-1'); 162.8 (C-4'); 164.8 (COO); 184.3 (COAr).

*Ethyl 3-(3,4-dimethoxybenzoyl)-pyrrolo[2,1-a]isoquinoline-1-carboxylate* (**4r**). Light brown crystals, m.p. 174–176 °C; Yield 69%. Anal. Calcd. C_24_H_21_NO_5_: C 71.45; H 5.25; N 3.47. Found: C 71.78; H 5.57; N 3.79. FT-IR (cm^−1^): 1184, 1266, 1458, 1515, 1625, 1709, 2957. ^1^H-NMR (CDCl_3_) 1.40 (t, 3H, *J* = 7.1 Hz, Me); 3.97, 3.99 (2s, 6H, 2MeO); 4.37 (q, 2H, *J* = 7.1 Hz, CH_2_); 6.98 (d, 1H, *J* = 8.3, H-6'); 7.26 (d, 1H, *J* = 7.4 Hz, H-6); 7.48 (d, 1H, *J* = 2.1, H-3'); 7.54 (dd, 1H, *J* = 8.3, 2.1 Hz, H-5'); 7.84 (s, 1H, H-2); 7.61–7.67 (m, 2H, H-8, H-9); 7.72–7.78 (m, 1H, H-7); 9.52 (d, 1H, *J* = 7.4 Hz, H-5); 9.80–9.85 (m, 1H, H-10). ^13^C-NMR (CDCl_3_) δ: 14.5 (Me); 56.1 (2MeO); 60.5 (CH_2_); 110.1 (C-1); 110.3, 112.3 (C-2', C-5'); 115.2 (C-6); 123.6, 124.8, 130.5, 132.5, 136.7 (C-3, C-6a, C-10a, C-10b, C-1'); 123.8 (C-6'); 125.1 (C-5); 126.7 (C-7); 127.7, 129.1 (C-8, C-9); 128.1 (C-10); 129.2 (C-2); 149.1, 152.7 (C-3', C-4'); 164.7 (COO); 184.7 (COAr).

## 4. Conclusions

In conclusion, new pyrrolo[2,1-*a*]isoquinolines were obtained by a simple one-pot three component cycloaddition reaction starting from readily available materials. The structures of the new compounds were assigned by IR and NMR spectroscopy. The regioselectivity of the cycloaddition was deduced on the basis of ^1^H-NMR data. The reaction is of potential interest due to importance of obtaining combinatorial libraries of compounds and due to the interest shown in the biological activity of compounds containing pyrrolo[2,1-*a*]isoquinoline skeletons. 
